# Perceptions, Experiences, and Challenges of Physicians Involved in Dementia Care During the COVID-19 Lockdown in India: A Qualitative Study

**DOI:** 10.3389/fpsyt.2020.615758

**Published:** 2021-01-20

**Authors:** Debanjan Banerjee, Bhavika Vajawat, Prateek Varshney, TS Sathyanarayana Rao

**Affiliations:** ^1^Department of Psychiatry, National Institute of Mental Health and Neurosciences (NIMHANS), Bengaluru, India; ^2^Department of Psychiatry, JSS Medical College and Hospital, JSS Academy of Higher Education and Research, Mysuru, India

**Keywords:** dementia care, COVID-19, lockdown, healthcare workers, experiences, India

## Abstract

**Introduction:** With 5.3 million people living with dementia in India and the pandemic wreaking havoc, dementia care has faced unique challenges during the outbreak, with reduced healthcare access, travel restriction, long-term lockdown and fear of hospitalization. We explored the experiences and barriers faced by the physicians involved in dementia care during the lockdown period.

**Methods:** A qualitative approach was used with purposive sampling. After an initial pilot, 148 physicians were included in the study. They were virtually interviewed in-depth based on a pre-designed semi-structured questionnaire, in areas related to tele-consultations, attributes related to dementia care, challenges faced and way forward. Interviews were recorded, transcribed and thematically analyzed using Nvivo-10 software. Triangulation, peer debriefing and respondent validation were used to ensure rigor.

**Results:** The overarching categories that emerged were “Tele-medicine as the future of dementia care in India,” “people living with dementia being uniquely susceptible to the pandemic with a triple burden of: *age, ageism and lack of autonomy*” and “markedly reduced healthcare access in this population with significant mental health burden of caregivers.” The experiences of the physicians were categorized into their challenges during the lockdown period and perceptions related to specific facets of dementia care during the crisis. The general physicians expressed special “unmet needs” of dementia-specific training and specialist collaboration. Most of the participants perceived ambiguity related to the newly released telepsychiatry guidelines.

**Conclusion:** Resource constraints and pandemic burden are currently high. This study looks at the “voices” of those actively providing dementia care during the ongoing crisis and to the best of our knowledge, is the first one from India to do so. Concurring with their experiences, PwD and their families are exposed to multiple vulnerabilities during COVID-19, need tailored care, especially at the primary healthcare level which includes general physicians. These relevant “voices” are discussed in light of the new tele-psychiatry guidelines and further optimization of dementia care in an aging India.

## Introduction

Older adults have been one of the most vulnerable populations during the Coronavirus 2019 (COVID-19) pandemic. Besides being exposed to the physiological risks of infection and increased fatality, it is further compounded by frailty, medical comorbidities, polypharmacy and pre-existing pulmonary complications (1). Furthermore, they are also prone to the persistent psychosocial offshoots of the pandemic including grief, isolation, loneliness, depression, anxiety and sleep disturbances (2). Among the elderly, people living with dementia (PwD) have been especially affected during the pandemic and consequent lockdown, with a multitude of factors contributing to the same. Lack of cognitive stimulation, mobility restriction, isolation, worsening of behavioral and psychological symptoms of dementia (BPSD), enhanced confusional states, increased chances of delirium, reduced adherence to precautionary measures, increased risks of abuse and institutionalization are some of the many factors contributing to the worsening of their overall health and cognitive status, and thus the consequent frailty (3, 4). The world is aging fast and it is projected that 1 in 5 people from the low and middle-income countries (LMIC) are going to be above 60 years of age. With an increase in older adults, there would be a proportionate increase in dementia prevalence, with a 5–7% projected rise in India and China. The absolute number of PwD is estimated to double by 2030, and treble by 2050, especially in the sub-continent (5). With a present 5.3 million dementia cases in the country, huge mental health gap (mhGAP), inadequate penetration of telepsychiatry and prolonged lockdown and economic downfall due to COVID-19, India has been facing unique challenges with dementia care. Marginalization, human rights deprivation, reduced healthcare access, increased symptoms of BPSD, social segregation and abuse have been reported sporadically among PwD in India during the last 6 months (6, 7). Systematic research in this area is still lacking. With a paradigm shift of mental healthcare delivery to virtual platforms, it is vital to understand the experiences and challenges faced by the nation's physicians while providing dementia care.

In general, physicians and other healthcare workers have faced unique plight during the pandemic. Studies have shown increase in stress, burnout, absenteeism, and stigma especially among the frontline workers (8, 9). Increased rates of depression, anxiety, sleep disturbances and post-traumatic stress have been reported among the physicians in a recent systematic review, especially in the developing countries with limited resources and increased COVID-19 burden (10). India is one of the low and middle-income countries (LMIC) that has one physician per 1,456 people as compared to the 1:1,000 ratio recommended by the World Health Organization (WHO) (11). It is also one of the countries with highest pandemic case load and due to the heterogeneity of the population, physicians in all specialties have faced significant challenges in delivery of adequate healthcare services during these crisis times. Even though differences have existed between the Indian Medical Association (IMA) and the Indian Government related to the COVID-19 policies for physicians, the administration has attempted several constructive steps in this regard (12). Healthcare resource building, ensuring medical safety for the physicians, staffing guidelines and timely payments, training in tele-consultations and round-the-clock psychological support are some of these measures. The Ministry of Health and Family Welfare (MoHFw), Government of India has mentioned about training guides for all level of healthcare workers, salary insurance, specific protocols for management of COVID-19 cases, testing and rational use of Personal Protective Equipment (PPE) in its officially released “measures” to ensure safety of healthcare workers (13). Nevertheless, systematic assessment of their daily challenges, taking into account their perspectives while policymaking and evaluating their psychosocial distress are sub-optimal that affect the implementation of the above-mentioned measures. The rise in violence, stigma, discrimination and dissatisfaction among the medical fraternity and prevalent misinformation in the media resonate the same (12). Gauging the situation, the WHO has collaborated with the Indian Government in training health workers and paramilitary forces in pandemic-specific measures using training of trainers (ToT) approach. The covered areas are epidemiology of COVID-19, bio-medical waste management, triage, mock drills, management of cases and peer-support for emotional well-being (14). While dealing with neurocognitive disorders itself is a challenging task during the pandemic, physicians in geriatric care face this “dual burden” of personal career-related adversities as well as the difficulties in caring for PwD during the pandemic-crisis. With this in the background, this study attempted to explore the “voices” of health care workers (HCW) at multiple sites in India with relation to consultations and care for PwD and their families.

## Methods

The study adopted a qualitative design with a constructivist approach and was approved by the JSSAHER Institutional Ethics Committee (JSSAHER University, Mysore) in March 2020. A “constructivist” paradigm as opposed to a positivist approach enables the researcher to stay “hypothesis-free” and conduct the work with the possibility of “multiple truths.” This is especially important in studying public perceptions as they cannot be statistically scaled or quantified and hence an *a-priori* hypothesis will be redundant in this case. It also helps in building an empathetic and collaborative relationship with the study participants, which is vital in qualitative methodology (15). A semi-structured interview guide was designed comprising of open-ended questions based on detailed discussion among the researchers, their clinical experiences related to the ongoing challenges of dementia care and existing literature on the challenges of HCW. It was piloted on eight participants initially and subsequently refined. Using professional connections and the directory available from the Indian Psychiatric Society (IPS), the physicians were contacted through email. Both snowballing and purposive sampling were used. The initial email asked if the particular physician was involved in consulting PwD during the lockdown period and were providing virtual consultations as well. These two conditions were necessary for inclusion in the study. Also, the mail explained the objectives and purpose of their study and sought their electronic informed consent. All participants provided explicit consent for participation in the study. The researchers ensured that the participation is well-distributed among the specialties (psychiatrists, neurologists, general physicians) who are involved in dementia care, the area and set-up of practice, age, gender and years of experience. This was again obtained through purposive and representative sampling. Physicians involved in long-term dementia care facilities were excluded as the patient profile, consultation patterns and physician engagement would have a totally different profile in that case and could potentially dilute the overall study results.

Though initially aimed at a multi-site Indian study, this particular paper looks at participants from various states of Southern India. The study was conducted between April–June 2020 when India was undergoing complete lockdown due to COVID-19. The actual interview was held virtually over Zoom/Google Meet over 1–3 sessions after obtaining consent. The first author, who was trained and certified in qualitative research methodology, conducted the interviews in Hindi and English. Each session lasted for an average of 102 ± 10.5 min. The semi-structured interview guide was used with open-ended probes, prompts and regular memo writing which would be later used for analysis (Box 1). The questions were aimed to explore their experiences, perceptions, and challenges related to dementia care consultations, with special emphasis on virtual service delivery. The interviews were recorded with consent and the responses transcribed and translated verbatim with back-translation with a researcher with bi-linguistic proficiency.

Box 1 **Semi-structured interview guide used for the study.**

- How has dementia care been different from you during the COVID-19 related lockdown?
- How do you feel about tele-consultation for people living with dementia and their caregivers?
- Please describe the facilitators and barriers related to virtual consultations for dementia care.
- How were the challenges in dementia consultations different from the pre-COVID times?
- What were the concerns expressed by people with dementia and their caregivers during your consultations? How did you manage them during the lockdown times?
- How has clinical and psychosocial concerns in dementia changed during the ongoing crisis?
- How has your role as a physician involved in dementia care changed due to the pandemic situations?
- What have your personal challenges been? *(not included in the study)*
- Considering the uncertainties of the pandemic, how do you foresee dementia care in the post-pandemic aftermath: please describe.

***For the general physicians***

- What were the challenges in “primary dementia care” that you have faced and what were your perceived “unmet needs”?
- How has primary dementia care been different during COVID-19 times?
- How do you think your consultations for people living with dementia were different from specialist consultations?

### Analysis

Charmaz's applied thematic analysis was used for the study (16). Data from the participants was generated through the above-mentioned semi-structured interview guide. Each interview was read word-by-word and coded (initial codes followed by clustering of codes to form “focused codes” and finally the mutual relationship between codes called axial coding). The coding was done by two independent researchers (first and fourth authors), who are trained in qualitative research. This was accompanied by a process of memo analysis and constant comparison back-and-forth with the coded data and the original transcripts for the rigor. The responses were analyzed in context and framework and the final hierarchy of themes and categories were reached only after rigorous discussion and brainstorming by all the researchers. Considering the voluminous amount of qualitative data, NVivo 10 software was used to organize and aid the analysis. However, each dataset was still manually coded for “immersion in data” and context which are important factors in such analysis. Nvivo 10 necessarily helps in storage, codification, organization and categorization of the qualitative data. It also facilitates the analysis, but the steps and process of coding and constant comparison have to be manual and can only be assisted by the software. Thematic saturation was achieved with 138 participants, however, 10 more were interviewed for super-saturation. After the initial invitation, 45 physicians hadn't responded and 23 did not consent for the study. Among the latter, majority mentioned lack of time while some others did not provide a reason for unwillingness to participate. Rigor was enhanced by triangulation in analysis, peer debriefing and respondent validation (where the initial results after the first round of analysis were presented to 50% of the sample and their inputs were sought about whether the results represented their “voices”) (17). The entire process of analysis was completed in about 2 months.

## Results

The terms physicians, general physicians (GPs) and HCW have been used interchangeably. The socio-demographics of the participants and their responses toward the interview are presented in Table 1. The mean age of the participants was 39.2 ± 5.3 years and the mean years of experience was 10.2 ± 2.4. The main categories and themes are summarized in Table 2. The results are divided mainly into the challenges faced related to dementia-case consultations during the lockdown, their experiences about various attributes of dementia care during that period, specific perceptions related to virtual (tele/video) service delivery and finally the specific themes that emerged related to the GPs. The authors agree that the GPs form a heterogeneous population and are bound to have different requirements and challenges with regards to dementia care, irrespective of the pandemic. However, considering the scarcity of specialist services in a developing country like India, GPs form the backbone of health care attending to most first consultations of neuropsychiatric disorders. Hence, the authors included them in the study which wanted to explore the experiences of any physicians dealing with PwD. Nevertheless, the detailed results related to the experiences of the GPs, their challenges and unmet needs will be presented in a separate paper. The overarching themes across all three specialties and irrespective of any other attributes included the recognition of PwD to have a “dual” vulnerability during the COVID-19 related crisis, concerns about their reduced access to care and perceived utility of “tele-medicine” as a promising platform for dementia care even in the post-pandemic aftermath. Most of the participant physicians shared their own “stress” as well during the lockdown crisis (though that was not a part of the study), welcomed the interview and felt that sharing their experiences especially related to dementia care was cathartic for them as well.

**Table 1:** Socio-demographics of the study participants (*n* = 148).

**Attribute**	**Types**	**No. (%)**
States	Karnataka Tamil Nadu Andhra Pradesh Kerala	68 (45.9) 31 (20.9) 20 (13.5) 29 (19.5)
Specialty	Psychiatrists Neurologists General Physicians	66 (41.9) 32 (21.6) 50 (33.7)
Age (years)	25–35 35–45 45–55 >55	49 (33.1) 56 (37.8) 25 (16.9) 18 (12.2)
Gender	Male Female	80 (54.0) 68 (46.0)
Area of practice	Urban Semi-urban Rural	72 (48.6) 30 (20.3) 46 (31.1)
Set-up of practice	Government Private (solo) Private (organization)	53 (35.8) 75 (50.7) 20 (13.5)
Experience (years)	0–5 5–10 >10 years	23 (15.5) 52 (35.1) 73 (49.3)
Modes of consultation during the lockdown	Only telephonic Only video Both telephonic and video Virtual and in-person	7 (4.7) 21 (14.1) 80 (54.1) 40 (27.0)
Opinion about the study interview	Useful Not useful Neutral Preferred not to say	115 (77.7) 10 (6.7) 15 (10.1) 8 (5.4)

**Table 2:** Categories and themes from the analysis (*n* = 148).

**Categories**	**Themes (% response)**	**Verbal excerpts of participants**
Challenges	• Virtual cognitive assessment in PwD (91%) • Worsening of behavioral and cognitive problems (90%) • Fear and stigma about in-person care (62%) • Availability of medications (43%) • Lack of patient advocacy: the establishment of therapeutic rapport (95%)	• “*It was indeed very difficult to apply scales over phone/video. The connection kept breaking and the patient could hardly comprehend what is being asked for”* • “*Most patients felt “locked inside” their rooms in absence of any stimulation and their agitation increased as well as memory disturbances”* • “*In most districts memantine and psychotropics were not available. Even though unmanageable at home, the family was scared of getting her admitted”* • “*I rarely spoke to the patient. Most virtual consults were on a proxy. It made engaging the patient all the more difficult”*
Experiences of dementia care during lockdown period	• Overuse of psychotropics to control BPSD (72%) • Reduction of autonomy and self-report (88%) • Perceived abuse from the patients (overt and covert) (69%) • Loneliness and isolation: contributing to behavioral worsening (96%) • Lack of basic amenities and Caregiver burnout/stress (85%)	• “*I found myself in a helpless situation. It was difficult to explain behavioral management to the family. I had to hike up the dose of risperidone multiple times”* • “*She complained of being locked in her room for the fear of getting infected and infecting others. Almost always she was tearful.”* • “*Most of my dementia patients are having increased depression, anxiety and sleep disturbances due to the prolonged loneliness and having no one to interact with”* • “*The husband was more concerned about the helper. The paid caregiver was off during the lockdown and he was even afraid to go to the market for daily necessities”*
Perceptions related to the online consultation	• Ambiguity related to the Telemedicine guidelines and legal implications (67%) • Ease of online consultation by the patients and reduced need for hospital visits (52%) • Enhanced access to healthcare for the patient population (69%) • Better maintenance treatment (47%) • Better cross-referral and discussion of other medical conditions (42%) • Ease of delivering basic psychoeducation involving multiple caregivers (62%) • Use of AI for cognitive stimulation (27%)	• “*We know about the guidelines being released. But its being hardly followed. I am not really aware of the legal responsibilities”* • “*Patients keep requesting for physical examination. It's so essential in neuropsychiatry. How will I prescribe without being sure…”* • “*It was easier to discuss with the cardiologist virtually, sometimes on the same platform. The need for physical referrals often delays it”* • “*For those who are doing well, they were quite satisfied with the tele-consultations”* • “*I didn't expect my rural clients to be so comfortable with video consults. They were quite tech-savvy, it surprised me. This is going to be the new norm”* • “*Demonstrating Lumosity/Brainwave/Cogmap was much easier. It was hands-on and certain families could implement it”*
Specific perceptions of the general physicians	• Challenges in assessment and screening of dementia (84%) • Overuse of medications (67%) • Lack of perceived satisfaction with the care delivery (52%) • Better expert referrals and discussion (42%) • Better learning: the need for virtual training (80%)	• “*Dementia has always been a challenge to us, especially early identification and conveying the diagnosis. The COVID times have made it even more difficult”* • “*Virtual consults are easy, but I often don't feel we are doing enough for the clients apart from prescribing the same meds”* • “*It is easier to virtually reach out for the district psychiatrist regularly. It helps a lot in understanding the cases. I hope it stays that way”* • “*Tele-medicine needs to specifically focus on training us in dementia care”*
**Overarching themes	• PwD considered as “especially vulnerable population”: Triple burden of “*age, ageism and autonomy”* (95%) • Decreased access to care (91%) • The perceived benefit of telemedicine as a “way of future” for dementia care (90%)	• “*I keep hearing about “vulnerable” populations during the pandemic. Well, these individuals share the burden of age, stigma and memory issues.”* • “*People living with dementia are in an impoverished state. They are most deprived of care among all the other psychiatric illnesses”* • “*At least others can express their need, they are sadly not able to “convey” that and are often neglected in this crisis, lonely and isolated…”* • “*It might have its limitations, but the way forward with limited manpower is telemedicine. It's the way to go, especially in developing countries.”* • “*Dementia care has a huge potential to be digitalized. Policies, guidelines and training need to be tailored accordingly”*

The verbatim excerpts supporting the generated themes are presented below (Table 2). Due to space constraints, only the pertinent ones are included in the manuscript. The detailed responses from the study are available from the authors on request.

## Discussion

### Tele-Medicine for Dementia Care: Pros and Caveats

Telemedicine aims at providing health care at distance to vouchsafe the interest of advancing the health of individuals and their communities (18). In a socio-culturally diverse and populated nation like India, virtual consultations can go a long way in reducing stigma, travel costs and enhancing healthcare access. This is in sync with the increased internet coverage in rural areas over the last decade and tripled smartphone usage during the pandemic (19). As highlighted in this study, physicians found themselves at a critical crossroads with tele-dementia care during the lockdown. This assumes a renewed importance for maintenance treatment in PwD, monitoring, management of BPSD and caregiver education, especially with the increased risk of complications and infection following hospitalization during the pandemic. Soares et al. (20) while discussing telecare for BPSD during COVID-19, describe it as a “viable tool” for monitoring clinical stability, however, cautions about cost, resistance to change, age of the patient, and technical challenges. Implementing remote memory clinics has also been recommended to help screening, digital cognitive training, pragmatic benefits of which can outlast the pandemic (21). HCP in this study rather admitted technical ease in consultations but were worried about the virtual assessments. The PwD may be technologically challenged, not comfortable with screen usage, compromised cognition may be further deteriorated due to the above. Vision and hearing impairment may compound the above leading to errors in assessment and compliance with instructions. Challenges in the cognitive assessment in general in the Indian population get more challenging virtually due to the multitude of cultural practices and vernacular languages, which predilect faulty assessment due to the dearth of language-sensitive assessment scales (22). Further, BPSD made cross-sectional assessments and monitoring for symptomatic severity problematic according to the physicians, leading to inadequate titration of psychotropic dosages.

Along similar lines, the assessment of physical health status was mentioned by many as a hurdle, especially in non-AD and those with medical co-morbidities. This increased the frequency of adverse effects due to psychotropic use in patients with dementia. The problem of patient's autonomy was a major “highlight” based on the current rights-based model and person-centered approach. According to our study, physicians were concerned about tele-care vicariously promoting proxy consultations, which mostly the patient's voices staying “unheard.” This can have a significant impact on dementia care during an already existing biopsychosocial crisis.

The newly released telemedicine and telepsychiatry guidelines 2020 (23), though helpful standards are still a long way from translating into actual clinical practice. The lack of awareness thereof and legal implications consequent to violation of the same is a harsh reality, as reflected by the “perceptions” in our study. Most anti-dementia drugs and anti-psychotics come in the “list B” of these guidelines and can only be given on follow-ups, which leads to difficulty in decision-making during the first consults (23). In the guidelines, a tele follow-up consultation is defined as “*patient consulting with the same psychiatrist within 6 months of his/her previous in-person consultation”* and the present consultation is for the continuation of care of the same clinical condition. Pragmatically, this is not always possible due to the individual preferences of the patients and professional availability of the physicians. Hence, prescribing medications for dementia patients over telephonic follow-ups was an “ambiguous” area for most psychiatrists. More than half of the neurologists and two-thirds of general physicians in our sample were not aware of the newly released guidelines. One of the possible reasons could have been that the guidelines were released during the time of the present study and were not yet popularized when the interviews were being conducted. Besides, there was also confusion about the applicability of the “same condition for continuation of care” as the initial visit necessary for telephonic follow-up. For example, many patients with dementia opted for review consultations to deal with the associated medical comorbidities or familial issues, which were not directly linked to the cognitive disorder *per se*, for which they had initially consulted. Another important concern raised by our participants was related to the consent of the patients and advanced directives. These tend to emerge as vital issues in dementia care, especially in areas of palliative care, end-of-life management and physician-assisted suicide (24). Though the Telepsychiatry guidelines explicitly discuss about documenting patient's consent, comfort to speak about his/her issues in the presence of family members and following the advanced directives as laid down by the patient: more than 90% of all our participants were not clear about the exact provisions to be followed. However, neither these guidelines nor the Indian Mental Healthcare Act (MHCA), 2017 specifically address the end-of-life concerns and related medico-legal issues in dementia care. The need for multiple online consultations for prescription along with the unavailability of medications during the lockdown in rural pockets, delayed the initiation of treatment. Though some doctors mentioned the use of AI for home-based cognitive exercises, they were limited to the urban areas and were themselves trained in the same. Most of our participants mentioned resistance to the use of “digital interventions” by the caregivers apart from basic consultation. The advocacy of online or digitally-assisted cognitive training and rehabilitation for dementia patients was 67, 31, and 15% in the psychiatrists, neurologists and general physicians in our study, respectively.

In a yin-yang world, the advantages of telemedicine are also many which were resonated in our study. In India, respite centers for PwD are their own homes with primary caregivers usually being family members. Thus, with the advent of tele-medicine the synchronous sensitization and education of the patient along with multiple caregivers including paid attenders, has not only becoming easier but also economical. The community approach of facilitating availability, affordability, accessibility, acceptability, continuity is further enshrined on tele-consultations (25). Tirthalli et al. (26) have rightly pointed out the difficulty in in-person consultations with face-masks, as a mental status examination is much more about “non-verbal cues” rather than clinical interviewing. This need for “unmasking of mind” becomes all the important in dementia care, where the expressive and comprehensive deficits tend to be further compromised “behind the mask.” Hence, the scope of tele-consultations. Multiple caregivers could be involved in the “behavioral assessment and analysis” in our study, which helps the treatment of BPSD. This is in line with our participants looking up to telemedicine as the “future of integrated dementia care” that involves easy cross-specialty referral. With a rapidly aging sub-continent with increased dementia-burden and limited, localized specialized resources, tele-care has been viewed by the physicians as a “dual-edged” path.

### Dementia Care During the Ongoing Pandemic

The ongoing pandemic has a bidirectional effect on dementia. The chances of cognitive impairment including delirium are higher in patients with COVID-19 who are already having dementia (3). Besides, the lack of understanding, comprehension, following social distancing protocols and hand hygiene predisposes PwD to the outbreak. The social isolation and loneliness perceived by our HCW can potentially worsen both the cognitive symptoms and BPSD (27). Added to that, was the “perceived abuse and prejudice” that was reported by the care-providers and they felt the “virtual medium” as a barrier for appropriate psychosocial interventions in this regard. Abuse and ageism related to dementia have been reported to be on the rise in developing countries, which can further impair the quality of life in PwD (1, 7). During the lockdown restrictions, the loss of autonomy and “coercive care” were also reported by our participants which was often relayed by the caregivers themselves. Similar “helplessness and benevolent restrictive” measures were reported by the caregivers in another qualitative study from India during the COVID-19 situation (28). These can hamper the dyadic relationship between PwD and their caregivers, consequently increasing the vicious cycle of elder abuse (29). Especially, in the Indian context where family members have the onus of caregiving and dementia is considered to be a part of normal aging. The above “red flags” were mentioned as reasons for a delay in diagnosis and help-seeking during the lockdown, with the need to use an inadvertent dose of psychotropics for “immediate relief” of both patients and caregivers, however increasing the risk of adverse effects and potentially against the “first-line” non-pharmacological management guidelines for BPSD (30). “The start low go slow” approach was reported as “better said than done” by our physicians, as medicines with sedative properties were self-titrated by the caregivers. The necessary need for the caregivers to identify signs of deterioration, adverse effects, medical complications were also hampered by the time and bandwidth-limited digital consultations during the lockdown. Due to the rising COVID caseload in India and predominant deaths in the older age-group (31), even patients needing hospitalizations were requested to be managed online, which was a major perceived challenge by the HCW. Significant caregiver burden emerged, mostly due to BPSD, uncertainty, socio-economic issues, and lack of paid caregivers, which have also been reported in studies from other developing countries (32). They could be better dealt with tele-consultations, as per the physicians involved in dementia care. Expectedly, caregiver interventions have the potential to improve the overall health of PwD as well.

Another important factor is the fear of COVID-19 that reduces healthcare access in older adults and their families, as well as builds up stigma for hospital visits. Both these factors can impair dementia care together with the imbalanced healthcare resource allocation in many countries. The physicians in our study reported difficulty in maintaining in-person appointments with significantly reduced compliance even when the out-patient services were running. Similar findings have been reported from Italy by Spalletta et al. (33) where 66.7 and 77.4% of patients had missed out on their first and follow-up visits, respectively, during the first wave of the pandemic, mainly due to the administrative restrictive measures imposed to curb the viral spread. The authors highlighted enhanced access of healthcare by PwD and their caregivers as a “compelling priority” to prevent burden of the gradually re-opening healthcare clinics. Van Jaarsveld (34) mentions about the “digital divide” that has impacted the elderly population and their healthcare system the most and focuses on digital literacy as an important facilitating tool for optimal utilization of tele-medicine services. The same was resonated by most of our physicians supporting digital-training of all stakeholders involved in dementia care (patients, caregivers, healthcare workers, and administrators) and highlighting the importance of digital health-education to improve public awareness. Service delivery and resource allocation for dementia care were also concerns raised by the participants. Decreased community support, primary healthcare facilities and social networking were reported among Spanish older adults in a recent study (35). Triage during pandemic care, especially critical care beds and ventilators, tend to be vital, more so in settings with limited resources. This assumes paramount importance in older adults, who are more susceptible to both mortality and morbidity due to COVID-19. A cross-sectional online survey done in Canadian physicians reported “presence of dementia” and likelihood of survival as two important factors in deciding healthcare resource allocation during the pandemic (36). The participants of the study were unsure about the required social support to organize and implement the necessary resource allocation. This has been replicated in Indian studies as well where the physicians have felt underprepared to make appropriate health-triage decisions especially in older adults, and perceived lack of emotional support (37). In our study, the general physicians reported this concern much more, especially those working in primary and sub-urban healthcare settings with limited resources. In many cases, patients affected with COVID-19 with comorbid dementia were considered to be the “last priority” due to therapeutic nihilism. This would affect both the course of dementia as well as the infection, impairing the overall quality of life. The neurologists in our study preferred psychiatric referrals for psychosocial interventions and focused more on pharmacotherapy for the control of BPSD.

### The “Triple Burden” During COVID-19: Age, Ageism, and Autonomy

There is a complex and dynamic interaction between an individual with dementia who has high dependency needs, living with various psychosocial adversities including the risk of abuse that is further complicated by the challenges posed by COVID-19 pandemic. This tetrad of “age, ageism, autonomy and COVID-19” expressed in our study seems to act synergistically in increasing the burden of PwD. Aging inherently poses several challenges and the risk of severe illness from COVID-19 increases with age. Eighty percent of the COVID-19 deaths in developed countries and 50% of the COIVD-19 deaths in India have been adults more than 60 years of age (38). COVID-19 has posed special needs of social distancing and self-isolation. In this highly dependable population requiring a physical form of care, this could lead to neglect, prejudice of ageism and also physical abuse which might go unnoticed by the physician over tele-consultations or go unreported. This is concerning with an already rising rate of elder abuse in India during the lockdown (39).

While the need for dependency and care is acknowledged, our participants felt it essential to balance it by preserving the autonomy of the PwD. While autonomy is an ethical construct that demands the highest advocacy, studies have reported that autonomy restriction can further increase the behavioral problems related to dementia (40). Some of the important factors leading to abuse according to our study could be poor knowledge about managing BPSD among the caregivers, caregiver burden, restriction of autonomy, and limitations of virtual consultations. Various studies have reported the successful use of patient-tailored, home-based psycho-educational interventions delivered *via* user-friendly online platforms to handle behavioral disturbances in PwD and reduce mood as well as anxiety symptoms among their caregivers which have resulted in an improved quality of life of the dyad (41).

### Barriers for the General Physicians in India

India has 0.75 psychiatrists per 100,000 population compared to six psychiatrists per 100,000 population in high-income countries (42). Also, there is only one neurologist catering to a population of one million in India. Dementia care is majorly dealt with by primary care set-ups in communities (43). Studies have reported that primary care physicians face challenges in diagnosis, and a majority of the PwD go unrecognized (43). The existing treatment gap for dementia in India is estimated to be around 90% with scarce specialist resources (44). Previous studies have identified barriers such as lack of support for patients, caregivers, and physicians, time and financial constraints, stigma, diagnostic uncertainty, and concerns around disclosure of the illness in the diagnosis and management of PwD among the primary care physicians (45). Another study among primary care physicians identified challenges such as lack of confidence in neurocognitive evaluation, implementation of screening, interpretation of standard diagnostic procedures, and prognostication. Unique needs such as managing medical comorbidities, polypharmacy, behavioral and psychological symptoms further complicate the care of the PwD (46). This study highlights several of these challenges similarly faced by physicians as the previous studies that have been conducted worldwide and additionally identifies certain COVID-19 related unique concerns. While on one hand, some of the challenges faced by the primary care physicians have further accentuated during COVID-19, several areas are identified which appear to hold a promise. The physicians in this study have identified telemedicine as a potential tool to assist management of PwD including facilitation of cross-referral. They have however expressed dissatisfaction due to the perceived lack of training in terms of holistic management of a PwD and need to use “more sedative medications” to manage BPSD during COVID-19.

The recently released telepsychiatry guidelines also mention about the collaborative consultation between any healthcare workers and the psychiatrist, especially in community and custodial settings (23). These include primary care physicians, nurses and other allied healthcare professionals. It provides for such cross-consultations in custodial, correctional, community areas, and rehabilitation centers as well as during home-visits, medical camps, and primary healthcare establishments. While such provisions were welcome for dementia-care and psychiatric training by the general physicians, the challenges conveyed by our participants were excessive workload, lack of digital resources and good connections in primary healthcare centers, poor collaboration with the specialists and time-constraints. More than half of the general physicians in our sample reported time being a crucial component in the dementia-care as the “need for quick improvement in behavioral symptoms” and “waiting for specialist referral” often led to losing the patient to follow-up. This often lead to self-perceived “threat to their competence,” reduced confidence for dementia care and increased use of psychotropics in our participants. They revealed the need for “better guidance” and cross-collaboration with psychiatrists and neurologists to manage patients with dementia and their families, but most were unaware of the newly released guidelines that could facilitate the same. Given the dearth of trained health professionals who manage dementia, alternative options of capacity building, task shifting, training, and the use of digital mental health intervention are highly recommended. The recently launched ECHO project by the National Institute of Mental Health and Neurosciences (NIMHANS) in Dementia Care is one such promising step (47). Further exploration of the “unmet needs” of general physicians involved in primary dementia care will help address policies and programs. Adequate tele-training of primary HCW, cross-collaboration and specialist guidance might be more pragmatic in the “digital future of dementia care” as mentioned by the physicians in our study.

The present study being qualitative has its inherent limitations, one of which is limited generalizability. However, qualitative studies in general are not intended have widely generalizable findings, as the perceptions of individuals are contextual from a constructivist vantage point of research, where even a “single voice” matters. Also, our sample size is relatively larger for a qualitative study and we have tried our best to have a representative sample from various regions, area of practice, and age-groups through purposive sampling. The physicians were however only from South India, and cannot be considered to be a pan-Indian sample. The other possible limitation is the researchers' bias while coding the data, themselves being physicians and facing similar challenges. We tried to deal with this by “constant comparison” of the analyzed results with the verbatim excerpts from the participants. Also, at each level of coding, there was rigorous discussion among the researchers with independent coding by two researchers. Lastly, the study was conducted during the period of COVID-19 related lockdown and the associated psychosocial challenges could have colored the opinion of the physicians; however, that was one of the objectives of the study. Besides, we haven't categorized the results based on age, years of experience and speciality but we also didn't find any major differences in the perceptions/experiences based on these attributes. There were certain nuanced variations in the themes among the groups which are highlighted in the discussion. To summarize, even with these pragmatic limitations, the study was rigorous in design and analysis with the results being grounded in the “voices” of the participants.

## Conclusion

The pandemic has been an unprecedented crisis for vulnerable populations. The Indian Ministry of Health and Family Welfare (MoHFw) in its “Health Advisory for Elderly Population during COVID-19” has stressed the special needs of older people with cognitive impairment, their healthcare access and preservation of rights and autonomy (48). The newly released telemedicine guidelines will serve as an effective anchor for implementing virtual “dementia care,” provided the physicians are well-versed with it. Most of the 5.3 million PwD are in the semi-urban and rural areas of India, and tele-health even with its pragmatic “caveats” can reduce travel costs, enhance access to care, decrease infection risks during a pandemic and improve specialist consultations. This study looks at the “voices” of those actively providing this healthcare and to the best of our knowledge, is the first one from India to do so. Concurring with their experiences, PwD and their families are exposed to multiple vulnerabilities during COVID-19, need tailored care, especially at the primary healthcare level which includes general physicians. Addressing the unmet needs of the physicians involved in dementia care during this time, improvisation of virtual cognitive assessments and cognitive rehabilitation, and further research into the systematization of digital platforms for such purposes can shape practice and policies even in the post-pandemic aftermath and during such futuristic crises. The newly released telepsychiatry guidelines have the potential to form an effective anchor for the same, and subsequent research into dementia care in India needs to explore the understanding, implementation and feedback related to the same.

## Data Availability Statement

The original contributions generated for the study are included in the article/supplementary material, further inquiries can be directed to the corresponding author/s.

## Ethics Statement

The studies involving human participants were reviewed and approved by JSS Academy of Higher Education and Research. The patients/participants provided their written informed consent to participate in this study.

## Author Contributions

DB, PV, and BV were involved in data collection, curation, organization, and drafted the manuscript. DB and TR were independently involved in analysis, and also responsible for editing and supervising. All authors have read and approved the final version of the manuscript, and involved in the study conceptualization, intellectual content, and design.

## Conflict of Interest

The authors declare that the research was conducted in the absence of any commercial or financial relationships that could be construed as a potential conflict of interest.
